# Reducing Self-harm in Adolescents. An individual participant data meta-analysis (RISA-IPD): systematic review protocol

**DOI:** 10.1136/bmjopen-2021-049255

**Published:** 2021-05-03

**Authors:** Alexandra Wright-Hughes, Rebecca Walwyn, Judy M Wright, Amanda Farrin, Peter Fonagy, Dennis Ougrin, Daniel Stahl, David Cottrell

**Affiliations:** 1 Complex Interventions Division, Clinical Trial Research Unit, Leeds Institute of Clinical Trials Research, University of Leeds, Leeds, UK; 2 Academic Unit of Health Economics, Leeds Institute of Health Sciences, University of Leeds, Leeds, UK; 3 Research Department of Clinical, Educational and Health Psychology, University College London, London, UK; 4 Department of Child and Adolescent Psychiatry, Institute of Psychiatry, Psychology and Neuroscience, King's College London, London, UK; 5 Department of Biostatistics and Health Informatics, Institute of Psychiatry, Psychology and Neuroscience, King's College London, London, UK; 6 Division of Psychological and Social Medicine, Leeds Institute of Health Sciences, University of Leeds, Leeds, UK

**Keywords:** child & adolescent psychiatry, suicide & self-harm, clinical trials

## Abstract

**Introduction:**

Up to 10% of adolescents report self-harm in the previous year. Non-fatal repetition is common (18% in 1 year), death from any cause shows a fourfold and suicide a 10-fold excess. Despite the scale of the problem, there is insufficient evidence for effective interventions for self-harm. Those who self-harm do so for a variety of different reasons. Different treatments may be more effective for subgroups of adolescents; however, little is known about which subgroups are appropriate for further study. This protocol outlines a systematic review and individual participant data meta-analysis (IPD-MA) to identify subgroups of adolescents for which therapeutic interventions for self-harm show some evidence of benefit.

**Methods and analysis:**

A systematic literature search was conducted in August 2019 (including Cochrane Library, Embase, trial registers and other databases). An update search is planned. Study selection will identify randomised controlled trials examining interventions to reduce self-harm in adolescents who have self-harmed and presented to services. Identified research teams will be invited to contribute data and form a collaborative group. Two-stage IPD-MA will be used to evaluate effectiveness of different therapeutic interventions compared with any active or non-active control on repetition of self-harm, general psychopathology, depression, suicidal ideation, quality of life and death. Subgroup analyses will identify adolescent subgroups in whom different therapeutic interventions may be more effective. Meta-regression will explore moderating study and intervention effects. Sensitivity analyses will incorporate aggregate data from studies lacking IPD and test the robustness of results to methods for handling missing data, within-study clustering, non-adherence and study quality.

**Ethics and dissemination:**

Ethical approval is provided by the University of Leeds, Faculty of Medicine and Health Ethics Committee (18-098). Outcomes will inform research recommendations and will be disseminated internationally through the collaborative group, a service user advisory group, open-access peer-reviewed publication and conference presentations.

**PROSPERO registration number:**

CRD42019152119.

**Strengths and limitations of this study**

- Much research in this field spans the adolescent and adult divide (ie, participants aged 14–25); individual participant data meta-analysis (IPD-MA) allows us to increase the pool of studies and participants through the inclusion of subsets of participants from partially eligible studies.
- IPD-MA will provide an increased sample size to permit meaningful subgroup analyses unavailable to individual studies; a more reliable estimate of the effect of therapeutic interventions for self-harm; and flexibility in analysis to handle sources of between-study and within-study heterogeneity.
- Obtaining agreements with primary authors of eligible studies to share data, development of formal data sharing agreements and the successful transition of data is the most likely obstacle to successful completion.
- Non-standardised baseline data may limit the available subgroup analyses and variation in the definition and follow-up periods for our primary outcome, repeat self-harm, requires careful derivation in the IPD-MA.
- Within IPD-MA, there is limited methodological guidance to account for treatment-related clustering; within-study clustering using standardised mean differences; and study, provider and patient-level mediators.

## Introduction

Self-harm is common in adolescents and a major issue of public health concern in the UK and globally.^1^ One in twenty 11–16 year-olds, and one in seven 17–19 year-olds, report having self-harmed or attempted suicide at some point; with rates in girls twice that of boys.^2^

Self-harm in adolescents has serious consequences; rates of death from any cause show a fourfold and suicide a 10-fold excess risk,^3^ and suicide is the second most common cause of death in 10–24 year-olds.^4^ Non-fatal repetition is common with 1-year rates of hospital reattendance at 18%.^5^

Any intervention that reduced self-harm would, as well as saving lives, result in significant reductions in family and peer distress. Effective interventions would also reduce significantly the cost to the health service in providing support for repeated self-harm.

A recent updated systematic review (25 studies, n=2962) found a small overall effect on repetition, with suggestive effect sizes for dialectical behaviour therapy, family-centred therapy, mentalisation-based therapy and interpersonal therapy.^6 7^ However, a single effective intervention to prevent repeat self-harm has not yet been identified.^6–8^ Published studies to date have often been small with mixed and varied heterogeneous samples.

Most published studies have been pragmatic and have recruited participants presenting to services without major restrictions on eligibility, in order to enhance generalisability. Those who self-harm do so for a variety of different reasons. It is therefore possible that there are subgroups of adolescents who respond better to all or some interventions.

We know that factors such as age, gender, lesbian, gay, bisexual, or transgender (LGBT) status, number of previous self-harm attempts, method of self-harm, psychiatric history and status (in particular depression^9^) carry increased risk for repetition. A recent National Confidential Inquiry into Suicide and Homicide indicated that of 285 suicide deaths that occurred in youths aged 10–20, 52% had a history of self-harm, while 58% expressed thoughts of suicide or hopelessness.^10^ This raises the possibility that either treatment and/or adolescent and family variables may predict better (or worse) outcomes and be important mechanisms underpinning effective interventions.

An individual participant data meta-analysis (IPD-MA) would provide more reliable estimates of the effects of therapeutic interventions for self-harm than conventional meta-analyses that rely on aggregated information and reported analyses,^11^ and would permit meaningful subgroup analyses previously unavailable to individual studies due to the increased sample size.

The *Reducing Self-harm in Adolescents: Individual Participant Data meta-analysis* (*RISA-IPD*) project aims to build on previous systematic reviews^6–8 12–14^ and conduct an IPD-MA of randomised controlled trials (RCT) of any control versus therapeutic intervention to reduce subsequent self-harm in adolescents who have already self-harmed and presented to services. The project objectives are to:

1. Conduct a systematic literature search and systematic study selection to identify relevant research teams and studies.
2. Invite identified research teams to contribute data to enable us to form a collaborative group and conduct IPD-MA.
3. Conduct IPD-MA to:
  1. Provide updated estimates of the pooled treatment effect of therapeutic interventions for self-harm compared with any non-active control.
  2. Identify subgroups of adolescents based on participant-level covariates, in whom therapeutic interventions are more effective.
  3. Explore moderating study and intervention effects.
4. Provide clearly defined research recommendations for future clinical practice and RCTs.

## Methods

### Study design

Systematic review and IPD-MA of RCTs of therapeutic interventions to reduce subsequent self-harm in adolescents who have already self-harmed and presented to services. A record for this IPD-MA can be found on PROSPERO.

### Eligibility criteria

#### Participants

All adolescents of any gender or ethnicity:

- Aged 11–18, where 18 is defined as up to the 19th birthday at the point of randomisation.
- Who have self-harmed at least once prior to randomisation and consequently presented to clinical services, where self-harm includes suicide attempt and excludes suicidal ideation without explicit self-harm.

No restrictions are placed on whether participants have comorbid mental or physical health conditions or intellectual disability.

#### Interventions

Any type of intervention, delivered by any type of care provider(s), one of the aims of which is to reduce subsequent self-harm. This includes psychological or pharmacological interventions, with/without individual, group or family involvement; delivery of social/service support; and interventions of any intensity including self-help. Prevention-based interventions not targeted specifically at adolescents who have presented to clinical services with self-harm and intensive inpatient-based interventions are excluded.

Evidence synthesis will be conducted by therapeutic intervention grouped by consensus of RISA-IPD clinical coapplicants (DC, DO, PF), according to the study intervention’s published descriptions, theoretical underpinnings, available supplementary material and manuals and is anticipated to follow:

- Cognitive–behavioural therapy.
- Dialectical behaviour therapy.
- Family therapy.
- Group therapy.
- Mentalisation, psychodynamic, cognitive analytic therapy.
- Multisystemic therapy.
- Pharmacological intervention.
- Problem solving, psychoeducation, support.
- Postcards, tokens, documents.

#### Controls

Any inactive (eg, treatment as usual, management as usual, placebo or attention control) or any active control.

#### Outcomes

##### Primary outcome

Repetition of self-harm. Defined as a cumulative binary outcome from randomisation to last available follow-up period within 3, 6, 12, 18 and 24 months postrandomisation.

The primary time period is 12 months postrandomisation. For the primary outcome, this will include studies where the follow-up assessment of self-harm took place between >6 and ≤12 months postrandomisation, with self-harm measured from randomisation.

Self-harm is defined as any form of intentional non-fatal self-poisoning or self-injury (including cutting, taking excess medication, attempted hanging, self-strangulation, jumping from height, running into traffic), regardless of suicidal intent. This includes definitions of non-suicidal self-injury commonly used by US researchers and suicidal behaviour where lack of intent is assumed by reference to the method of self-harm. Self-harm can be self-reported.

##### Secondary outcomes

- Time to repetition of self-harm.
- Pattern of self-harm repetition over time.
- General psychopathology: score on a self-report measure of emotional and behavioural problems.
- Depression: score on a self-report measure of depression.
- Suicidal ideation: score on a self-report measure of suicidal ideation.
- Quality of life: score on a self-report Quality of Life Scale.
- Death of adolescent.

Follow-up assessments will be grouped in the short term (up to 3 months postrandomisation), and at 6, 12, 18 and 24 months postrandomisation. Where studies include assessments beyond 24 months, data will be included where feasible and grouped as ≥24 months postrandomisation.

#### Setting/context

All countries of origin, any method of referral but ongoing intervention delivered in outpatient or community (school and voluntary sector) settings. This excludes intensive inpatient-based interventions as these are unlikely to be applicable to UK settings.

#### Studies

All RCTs, from the first available study, with any randomised design (eg, individual or cluster), length of follow-up and quality, in which data relating to self-harm or suicide attempts have been collected.

We will include studies in which only a subset of participants meet our eligibility criteria where we are able to obtain IPD for eligible participants, that is, studies with only a subset aged 11–18, or not all having self-harmed at least once prior to randomisation. Studies with less than 20 eligible participants will be excluded to ensure the logistical effort in obtaining, cleaning and organising the data is commensurate with the contribution of the data set to the analysis.

### Search strategy

A scoping search in 2018 identified five systematic reviews^7 8 12–14^ that identified 22 RCTs meeting RISA-IPD eligibility criteria (online supplemental material 1). However, assessment of the search strategies and inclusion criteria of these systematic reviews indicated eligible studies may have been missed if they were unpublished, recently published or contained <85% adolescents as participants.

10.1136/bmjopen-2021-049255.supp1 Supplementary data

Our search strategy seeks eligible RCTs by two routes to ensure exhaustive coverage of relevant systematic reviews, and retrieval of eligible studies missed or excluded by previous systematic reviews (online supplemental material 2):

10.1136/bmjopen-2021-049255.supp2 Supplementary data

- Literature search for systematic reviews of self-harm in adolescents to harvest potentially eligible cited RCTs.
- Literature searches for recently published RCTs. The publication date limit will be the date of the searches in the last systematic review that reports an exhaustive search. Our scoping searches indicate this is likely to be 2015. Also, literature searches for unpublished trials (no date restriction) and ongoing trials.

We will adhere to European Network for Health Technology Assessment search guidance.^15^ The information specialist and core team collaborated to develop Medline search strategies consisting of text words and subject headings for self-harm, suicide behaviours and adolescents. One strategy will be limited to systematic reviews and a second strategy will be limited to RCTs. The Cochrane Collaboration RCT filter is used to identify studies in Medline. The Medline search strategies are reproduced in full in online supplemental material 2. The systematic review search will be translated and run in Cochrane Database of Systematic Reviews, Embase 1947+, Epistemonikos, Medline 1946+, PROSPERO and PsycINFO 1806+. The RCT search will be translated and run in ClinicalTrials.gov, Cochrane Central Register of Controlled Trials, Embase 1947+, Epistemonikos, Europe PMC Grant finder, Medline 1946+, National Health and Medical Research Council (MRC, Australia), National Youth Mental Health Foundation (Headspace), ProQuest Dissertations & Theses A&I 1743+, PsycINFO 1806+, Web of Science Conference Proceedings Citation Indexes–Science and Social Science & Humanities 1990+, WHO International Clinical Trials Registry Platform. These results will be combined in an EndNote library and screened/selected using Covidence software. The search for RCTs will be repeated 9 months prior to the end of the project.

#### Review strategy/selection process

Studies will be identified using the search strategy above. Two reviewers (DC, AWH) will review titles and abstracts, referring to full text and a third nominated reviewer (RW) as necessary, to select:

1. Systematic reviews containing RCTs of interventions for self-harm in adolescents.
2. Trials (from the RCT literature search and those harvested from selected systematic reviews) using agreed eligibility criteria. The reference lists of included studies will be checked for further relevant RCTs.

### Data collection

#### Study-level data collection and risk of bias

Papers, full protocols and statistical analysis plans will be obtained following enquiries to RCT chief investigators and statisticians, and data extracted on study quality (at the outcome and study level) by two reviewers (DC, AWH); authors will be contacted for further information as required.

The quality of identified eligible studies (ie, those contributing IPD and/or aggregate data) will be evaluated independently by at least two reviewers (DC, AWH) using the Risk of Bias 2 assessment tool.^16^ Criteria cover random sequence generation, allocation concealment, blinding of participants, outcome assessment and missing data. We will further evaluate additional sources of bias common to complex intervention RCTs (eg, allocation of therapists to participants, therapist-level missing data). Each domain will be judged as contributing to a low, high or unclear risk of bias.

Assessment will take place following identification of eligible studies based on published papers and protocols irrespective of the availability of IPD. Further assessment will be conducted for included studies after IPD are obtained, to allow data quality to be assessed in more detail. Studies will be included in the primary analysis regardless of study quality and adjusted for in planned meta-regression; sensitivity analyses will exclude studies judged as having an unclear or high risk of bias.

#### IPD collection

As recommended by MRC and the Cochrane Collaboration^17^ we will invite senior authors of each eligible study to join a study collaborative group. Multidisciplinary, cross-cultural membership of the collaborative group will ensure the data used are properly understood and used appropriately in the analysis to provide a more global and balanced interpretation of the meta-analysis results. Regular, informal contact between the study team and collaborators is expected as questions arise about individual studies. We will invite members of the collaborative group to be involved in and comment on drafts of primary publications and to be named as authors of those publications.

We will contact study authors of eligible studies to seek the following IPD:

- Baseline participant demographic and clinical data: age, gender, ethnicity, intellectual disability, LGBT status, history of abuse, looked after children, self-harm method, number and timing of previous self-harm attempts, comorbid psychiatric conditions (depression, borderline personality disorder and unemotional/callous traits, eating disorders, anxiety disorders).
- Details of therapeutic intervention: attendance, session frequency and duration, intensity, overall duration, therapist details.
- Outcomes: repetition of self-harm, suicide attempt: incidence and time to event, and associated data (severity, method, outcome, type of event—self-reported or clinical/hospital presenting). General psychopathology (emotional and behavioural problems), depression, suicidal ideation and quality of life collected using standardised validated measures at baseline and all available time points postrandomisation. Death of adolescent: incidence and time to event.

Data collection will be prioritised for the primary outcome repetition of self-harm.

Collection and collation of data will be coordinated by the team based at the Clinical Trials Research Unit (CTRU) at the University of Leeds. Participating study authors will be asked to provide pseudonymised (without identifying data) data sets in whatever format is convenient to them, along with data dictionaries, original statistical analysis plans and relevant statistical programming code, where possible. Data sharing and transfer will be subject to formal data sharing agreements signed by the University of Leeds and collaborating institutions.

We will make regular contact with study authors throughout the project and continue to seek to reach agreement with study authors to share data up to within 2 months of the start of analysis in order to allow sufficient resource for management of the data. IPD from studies will be excluded (by necessity) if it is not possible to reach agreement with study authors to share data, or not possible to obtain good quality translations of non-English reports by this time.

#### Management of IPD

A copy of the raw data obtained from each study will be archived and saved in a restricted folder on receipt, prior to any modification of the data. Published statistics and analyses will be replicated where possible to identify the variables used, and to check the data to ensure accuracy and quality. Issues and discrepancies found will be raised and rectified with the study author. Individual study data sets will be reformatted to facilitate the merge across studies. Variables will be derived as required and the resulting data set locked for analysis.

All information collected during the course of the study will be kept strictly confidential. The CTRU will comply with all aspects of the 2018 Data Protection Act, which incorporates the European Union General Data Protection Regulation. At the end of the study, original data sets provided by collaborating trialists will be destroyed and the study data set securely archived at the CTRU for a minimum of 5 years.

### Patient and public involvement

A service user advisory group has been established comprising four young people, aged 14–16, who are current service users with a personal experience of self-harm. Involvement of the group includes:

- Educating and familiarising the group and their parents/carers with the research methods.
- Discussion of the research questions, ensuring these reflect the needs and priorities of patients and the public.
- Ensuring relevant subgroups of participants are investigated.
- Reviewing and commenting on the progress of the research, emerging research results, interpretation and relevance to young people and their parents/carers.
- Helping write the plain English summary of research outputs.
- Helping disseminate research outputs through social media, blogs and conferences.

### Data synthesis

#### Statistical considerations

Analyses will be conducted in accordance with current recommendations for IPD meta-analyses^18–23^ and will consider appropriate adjustment for baseline covariates (prognostic factors), handling of multiple treatment groups and control arms, missing data,^24–27^ repeated measures,^28 29^ timing of outcomes, randomisation, within-study treatment-related clustering^30 31^ and non-adherence.

Analysis populations will be based on the intention-to-treat principle, including all randomised participants in the groups to which they were randomised, regardless of withdrawal or protocol compliance.

#### Descriptive analysis

Study-level, arm-level, care provider-level and participant-level characteristics of included studies will be summarised. We will compare these characteristics to studies that were eligible but did not supply IPD, to determine if the IPD studies are a representative and unbiased sample of all eligible studies. Funnel plots of the reported effect size of included studies will assess potential publication bias.^32^ Outcomes of included studies will be summarised, as will adolescent-level and study-level moderators specified in subsequent analysis.

#### Missing data

Missing (outcome/participant/study level) data will be summarised, distinguishing between ‘systematically missing’ (missing for all participants within a study) and ‘sporadically missing’ data (incomplete data observed for at least some individuals within a study). We will assess missing data mechanisms by comparing characteristics of studies and participants with and without follow-up data. Missing data will be handled in each study separately using appropriate methods for dealing with missing data in an RCT.^24–27^

#### Effect measures

Where outcomes comprise binary data, logistic regression will be used to produce OR estimates of the effect of treatment; results will be re-expressed as risk differences and relative risks to aid interpretation.

Where sufficient time-to-event data are available for outcomes repetition of self-harm and death, Cox proportional hazards regression will be used to produce HR estimates of the effect of treatment; absolute differences will be estimated from the model at relevant time periods.

Linear regression will be used to estimate the absolute mean difference in treatment effect where outcomes comprise continuous data from the same scales; where outcomes comprise continuous data from different scales, we will estimate the standardised mean difference (SMD), standardised according to the total SD.^30 31^

#### Analysis of the pooled treatment effect (objective 3.1)

Analysis will be conducted for all primary and secondary outcomes separately for each group of studies categorised according to therapeutic intervention. Outcomes for each time period will be analysed separately; however, where possible, we will also conduct multivariate two-stage IPD meta-analysis to include all time periods in the same analysis model, to borrow strength across time periods.^28 29^

Analysis will be based on a two-stage approach using IPD to estimate the intervention effect in each study separately, followed by meta-analysis to pool aggregate results. Estimates of the intervention effect for the primary outcome, repetition of self-harm, will also be conducted using a one-stage IPD meta-analysis to explore whether estimates are consistent and robust to different estimation methods and modelling decisions.^19 21^

Stage 1 analysis using logistic, linear and Cox proportional hazards regression (as appropriate to outcome) will adjust for a few key participant-level prognostic factors defined before any analysis and restricted to strong prognostic factors that are available in all (or most) available studies. Analysis will also account for within-study clustering of participants (ie, cluster randomisation, treatment-related clustering) using appropriate methods for each study. Where treatment effect is expressed as the SMD, primary analysis will not account for within-study clustering due to the additional complexity arising from the presence of repeated measurements for the estimated variation on which to standardise outcomes.^28–31^

Stage 2 analysis will estimate the pooled treatment effect using the random effects approach, assuming a normal distribution of treatment effects across studies allowing for heterogeneity in treatment effect caused by different study characteristics. A fixed effects approach will be used where necessary for binary outcomes with rare events (<10%) and multivariate analysis including all time periods.^28 29^

Estimation will use restricted maximum likelihood (REML)^23^ for both one-stage and two-stage analyses using a pseudolikelihood approach for one-stage analysis of the binary primary outcome. CIs will be derived using an approach to account for uncertainty in variance estimates, such as the Hartung-Knapp-Sidik-Jonkmann^22^ approach in the two-stage analysis, and the Kenward-Roger or Satterthwaite approach following the one-stage analysis.^21^

After the meta-analysis, we will report estimated summary treatment effects, 95% CIs and 95% prediction intervals alongside forest plots with study-specific estimates of treatment effect. Heterogeneity will be assessed using the τ^2^ statistic (variability of the true effect sizes under the random effects model, estimated using REML) and the I^2^ statistic (proportion of total variability due to between-study heterogeneity).

#### Analysis of adolescent subgroups (objective 3.2)

Provided sufficient data are available, we will investigate whether treatment effects for groups of therapeutic interventions are consistent across adolescent subgroups. Analyses will be carried out on the primary outcome, repeated for secondary outcomes, either where specified a priori or where evidence of subgroup effects is found on the primary outcome to check for consistency. Subgroups will be included in analysis if represented in a sufficient number of studies; studies not collecting the moderator of interest will not be included. Adolescent subgroups will be agreed by the project management and collaborator group, and are likely to consist of:

- Age: as a continuous variable.
- Sex: male, female.
- Ethnicity: White, other.
- Identifies as LGBT or not.
- Autistic spectrum disorder: present, absent.
- History of abuse: yes, no.
- Presenting self-harm method: self-injury, self-poisoning, combined.
- Number and timing of previous self-harm attempts: one, two, multiple.
- Family dysfunction.
- Comorbid psychiatric conditions: depression, borderline personality disorder, unemotional/callous traits, eating disorders, anxiety disorders.

The two-stage *Analysis of the pooled treatment effect (objective 3.1)* will be extended to investigate adolescent-level subgroup effects separately for groups of studies categorised according to therapeutic intervention. The first stage will include the adolescent subgroup as a fixed main effect and moderator-by-treatment interaction. The second stage will pool the interaction effects in a random effects meta-analysis to account for between-study heterogeneity in the within-study moderator-by-treatment interaction.

IPD meta-analysis will increase the power to detect genuine subgroup effects. To ensure effects detected are not due to chance finding in a single study, subgroup effects will be examined for consistency across studies and presented in a forest plot.

#### Analysis of moderating study and intervention effects (objective 3.3)

The effect of therapeutic interventions may vary across studies due to additional sources of between-study heterogeneity. This includes clinical diversity due to variability in participants or intervention delivery (eg, number and duration of sessions) and methodological diversity due to variability in study conduct and design (eg, age of study, country, data collection and outcome measurement, risk of bias). Provided sufficient data and studies are available, meta-regression will explore potential clinical and methodological study-level moderators of treatment effect.

IPD random effects meta-regression will extend the two-stage meta-analysis used to obtain estimates of the pooled treatment effect (*objective 3.1*) and adolescent subgroups (*objective 3.2*). Potential study-level moderators will be included as fixed main effects in the stage 2 meta-regression model to investigate if there are any substantial differences in the effect of treatment by moderator. Forest plots depicting the individual study and pooled treatment effects for different levels of moderators will be presented.

Analyses will be carried out on the primary outcome separately for groups of studies categorised according to therapeutic intervention and will be repeated for secondary outcomes where evidence of subgroup effects is found on the primary outcome to check for consistency.

#### Sensitivity analyses

Sensitivity analysis will test the robustness of our conclusions for the analysis of the primary outcome. These will include methods for handling missing data,^24–27^ within-study clustering effects for SMDs,^30 31^ non-adherence and study quality.

#### Unavailable IPD data

Where IPD are not obtained for eligible studies, we will use two-stage meta-analysis to incorporate aggregate data from studies lacking IPD (using published sources).^33 34^ Analysis will explore the impact on estimates of the pooled treatment effects (*objective 3.1*) and moderating study effects (*objective 3.3*). Analysis of adolescent subgroups (*objective 3.2*) will be dependent on availability of suitable aggregate data.

#### Confidence in cumulative evidence

To evaluate the strength of the body of evidence, we will report findings alongside our risk of bias assessment for individual studies^16^ and an assessment of potential publication bias via funnel plot asymmetry.^32^

## Ethics and dissemination

Formal ethical approval for the project is provided by the University of Leeds, Faculty of Medicine and Health Ethics Committee (MREC 18-098). The project is sponsored by the University of Leeds (RG.PSRY.116370) and funded by the National Institute for Health Research Health Technology Assessment Programme in the UK following a commissioned call (grant number 17/117/11). The sponsor had no involvement in development of the protocol but along with the funder approved the proposal developed by the research team.

An independent Study Steering Group including independent clinical and statistical experts and a patient and public involvement representative will provide independent oversight of the project. Important protocol amendments will be documented, submitted to the funder and reported alongside study results.

We will use the collaborative group networks to disseminate our findings internationally. The results will be published in an open-access peer-reviewed journal and abstracts submitted to general child and adolescent mental health and ‘subject specific’ conferences. We will also work with our advisory group and the Young Person’s Mental Health Advisory Group (https://ypmhag.org/) to create user-friendly and relevant materials for dissemination and will partner with organisations such as Childline and YoungMinds to disseminate results on their websites.

## Supplementary Material

Reviewer comments
