# Supplementary material for: Reducing Self-harm in Adolescents. An individual participant data meta-analysis (RISA-IPD): systematic review protocol
Source: BMJ Open. 2021 May 3;11(5):e049255. doi: 10.1136/bmjopen-2021-049255 (PMC8098984; doi:10.1136/bmjopen-2021-049255)
Supplement: Supplementary data [file bmjopen-2021-049255supp001.pdf]

Supplementary material 1

Studies from scoping review and key published reviews meeting RISA-IPD eligibility criteria

Study	Year	Country	N	Age	Intervention	Control	Self-harm reported as outcome	Follow-up length (months)
**Studies meeting eligibility criteria. All participants to be included in RISA-IPD**								
Asarnow[1]	2017	US	42	11-18	CBT/ DBT informed family management	*TAU + Parent support	Yes	12
Cotgrove[2]	1995	UK	105	<16	Token plus assessment as usual	Assessment as usual	Yes	12
Cottrell[3]	2018	UK	832	11-17	Family therapy	TAU	Yes	18
Donaldson[4]	2005	US	39	12-17	Skills based treatment	Supportive treatment	Yes	6
Green[5]	2011	UK	366	12-17	Group therapy plus TAU	TAU	Yes	12
Hazell[6]	2009	Australia	72	12-16	Group therapy plus TAU	TAU	Yes	12
Mehlum[7]	2016	Norway	77	12-18	Brief **DBT	Enhanced TAU	Yes	18
Ougrin[8]	2013	UK	70	12-18	Therapeutic Assessment	Assessment as usual	Yes	24
Rossouw[9]	2012	UK	80	12-17	Mentalisation based treatment	TAU	Yes	12
Spirito[10]	2002	US	76	12-18	Problem Solving	TAU	Yes	3
Wood[11]	2001	UK	63	12-16	Group therapy plus TAU	TAU	Yes	7

Wright-Hughes A, *et al. BMJ Open* 2021; 11:e049255. doi: 10.1136/bmjopen-2021-049255

**Studies partially meeting eligibility criteria, not all self-harmed prior to randomisation. Eligible participants to be included in RISA-IPD**								
Asarnow[12]	2011	US	181	10-18	Family based CBT	TAU	Yes	2
Chanen[13]	2008	Australia	86	15-18	Cognitive Analytic Therapy	TAU	Yes	24
Esposito[14]	2011	US	40	13-17	CBT	Enhanced TAU	Yes	18
Huey[15]	2004	US	156	10-17	MST	Hospitalisation	Yes	12
King[16]	2006	US	289	12-17	Youth nominated Support plus TAU	TAU	Yes	6
King[17]	2009	US	448	13-17	Youth nominated Support v2, plus TAU	TAU	Yes	12
Pineda[18]	2013	Australia	48	12-17	Interactive psycho-education for parents plus TAU	TAU	Yes	6
**Studies partially meeting eligibility criteria, not all participants aged 11-18. Eligible participants to be included in RISA-IPD**								
Cooney[19]	2010	NZ	29	13-19	DBT	TAU	Yes	18
McLeavey[20]	1994	Ireland	39	15-45	Interpersonal Problem Solving	Brief problem-oriented approach	Yes	6
Robinson[21]	2012	Australia	164	15-24	Postcard plus TAU	TAU	Yes	18
Slee[22]	2008	Holland	90	15-35	CBT plus TAU	TAU	Yes	9

* TAU = Treatment as Usual

** DBT = Dialectical Behaviour Therapy

*** CBT = Cognitive Behavioural Therapy

Wright-Hughes A, *et al. BMJ Open* 2021; 11:e049255. doi: 10.1136/bmjopen-2021-049255

1. Asarnow JR, Hughes JL, Babeva KN, Sugar CA. Cognitive-Behavioral Family Treatment for Suicide Attempt Prevention: A Randomized Controlled Trial. *Journal of the American Academy of Child & Adolescent Psychiatry* 2017; **56**(6): 506-14.
2. Cotgrove A, Zirinsky L, Black D, Weston D. Secondary Prevention of Attempted-Suicide in Adolescence. *J Adolescence* 1995; **18**(5): 569-77.
3. Cottrell DJ, Wright-Hughes A, Collinson M, et al. Effectiveness of systemic family therapy versus treatment as usual for young people after self-harm: a pragmatic, phase 3, multicentre, randomised controlled trial. *The Lancet Psychiatry* 2018; **5**(3): 203-16.
4. Donaldson D, Spirito A, Esposito-Smythers C. Treatment for adolescents following a suicide attempt: Results of a pilot trial. *Journal of the American Academy of Child and Adolescent Psychiatry* 2005; **44**(2): 113-20.
5. Green JM, Wood AJ, Kerfoot MJ, et al. Group therapy for adolescents with repeated self harm: randomised controlled trial with economic evaluation. *British Medical Journal* 2011; **342**.
6. Hazell PL, Martin G, McGill K, et al. Group therapy for repeated deliberate self-harm in adolescents: failure of replication of a randomized trial. *Journal of the American Academy of Child & Adolescent Psychiatry* 2009; **48**(6): 662-70.
7. Mehlum L, Tørmoen AJ, Ramberg M, et al. Dialectical behavior therapy for adolescents with repeated suicidal and self-harming behavior: a randomized trial. *Journal of the American Academy of Child & Adolescent Psychiatry* 2014; **53**(10): 1082-91.
8. Ougrin D, Boege I, Stahl D, Banarsee R, Taylor E. Randomised controlled trial of therapeutic assessment versus usual assessment in adolescents with self-harm: 2-year follow-up. *Archives of Disease in Childhood* 2013; **98**(10): 772-6.
9. Rossouw TI, Fonagy P. Mentalization-based treatment for self-harm in adolescents: a randomized controlledtrial. *Journal of the American Academy of Child & Adolescent Psychiatry* 2012; **51**(12): 1304-13. e3.
10. Spirito A, Boergers J, Donaldson D, Bishop D, Lewander W. An intervention trial to improve adherence to community treatment by adolescents after a suicide attempt. *Journal of the American Academy of Child and Adolescent Psychiatry* 2002; **41**(4): 435-42.
11. Wood A, Trainor G, Rothwell J, Moore A, Harrington R. Randomized trial of group therapy for repeated deliberate self-harm in adolescents. *Journal of the American Academy of Child & Adolescent Psychiatry* 2001; **40**(11): 1246-53.
12. Asarnow JR, Baraff LJ, Berk M, et al. An emergency department intervention for linking pediatric suicidal patients to follow-up mental health treatment. *Psychiatric Services* 2011; **62**(11): 1303-9.
13. Chanen AM, Jackson HJ, McCutcheon LK, et al. Early intervention for adolescents with borderline personality disorder using cognitive analytic therapy: randomised controlled trial. *British Journal of Psychiatry* 2008; **193**(6): 477-84.
14. Esposito-Smythers C, Spirito A, Kahler CW, Hunt J, Monti P. Treatment of Co-Occurring Substance Abuse and Suicidality Among Adolescents: A Randomized Trial. *Journal of Consulting and Clinical Psychology* 2011; **79**(6): 728-39.
15. Huey SJ, Henggeler SW, Rowland MD, et al. Multisystemic Therapy Effects on Attempted Suicide by Youths Presenting Psychiatric Emergencies. *Journal of the American Academy of Child & Adolescent Psychiatry* 2004; **43**(2): 183-90.
16. King CA, Kramer A, Preuss L, et a. The Youth-Nominated Support Team for Suicidal Adolescents (version 1): a randomized controlled trial. *Journal of Consulting & Clinical Psychology* 2006; **74**: 199-206.

17. King CA, Klaus N, Kramer A, Venkataraman S, Quinlan P, Gillespie B. The Youth-Nominated Support Team-Version II for Suicidal Adolescents: A Randomized Controlled Intervention Trial. *Journal of Consulting and Clinical Psychology* 2009; **77**(5): 880-93.
18. Pineda J, Dadds MR. Family intervention for adolescents with suicidal behavior: a randomized controlled trial and mediation analysis. *J Am Acad Child Adolesc Psychiatry* 2013; **52**(8): 851-62.
19. Cooney E, Davis K, Thompson P, Wharewera-Mika J, Stewart J. Feasibility of evaluating DBT for self-harming adolescents: A small randomised controlled trial. Auckland, NZ: Te Pou o Te Whakaaro Nui. The National Centre of Mental Health Research, Information and Workforce Development, 2010.
20. McLeavey BC, Daly R, Ludgate JW, Murray CM. Interpersonal problem-solving skills training in the treatment of self-poisoning patients. *Suicide & Life-Threatening Behaviour* 1994; **24**(4): 382-94.
21. Robinson J, Yuen HP, Gook S, et al. Can receipt of a regular postcard reduce suicide-related behaviour in young help seekers? A randomized controlled trial. *Early Intervention in Psychiatry* 2012; **6**: 145-52.
22. Slee N, Garnefski N, van der Leeden R, Arensman E, Spinhoven P. Cognitive-behavioural intervention for self harm: randomised controlled trial. *British Journal of Psychiatry* 2008; **192**: 201-11.
