# Supplementary material for: Reducing Self-harm in Adolescents. An individual participant data meta-analysis (RISA-IPD): systematic review protocol
Source: BMJ Open. 2021 May 3;11(5):e049255. doi: 10.1136/bmjopen-2021-049255 (PMC8098984; doi:10.1136/bmjopen-2021-049255)
Supplement: Supplementary data [file bmjopen-2021-049255supp002.pdf]

Supplementary material 2

Systematic Review Search Strategy

Database: Ovid MEDLINE(R) and Epub Ahead of Print, In-Process & Other Non-Indexed Citations and Daily <1946 to June 20, 2019>

Search Strategy:

----

1 Self-Injurious Behavior/ (7200)

2 suicide/ or suicide, attempted/ (51983)

3 Drug Overdose/ (10369)

4 Self Mutilation/ (3180)

5 (selfharm* or selfinjur* or selfinflict* or "self harm*" or "self injur*" or "self inflict*").ti. (4978)

6 ((self or themsel* or onesel*) adj2 (aggress* or harm* or cutt* or immolat* or inflict* or injur* or mutilat* or poison* or damag* or destruct*)).ti. (7491)

7 (auto adj (aggress* or mutilat*)).ti. (75)

8 (automutilat* or "auto mutilation*" or autoaggress* or "auto agress*").ti. (222)

9 suicid*.ti. (40905)

10 (parasuicid* or para-suicid*).ti. (332)

11 ((deliberat* or intentional or intended) adj2 (overdos* or poison* or self poison*)).ti. (413)

12 (poison adj2 (deliberat* or intentional or intended)).ti. (3)

13 (overdos* adj2 (deliberat* or intentional or intended)).ti. (138)

14 NSSI.ti. (52)

15 (headbang* or head-bang*).ti. (70)

16 or/1-15 (80097)

17 Adolescent/ (1939543)

18 (teenage* or adolescen* or youth).tw. (304577)

19 young adult/ (749895)

20 (young* adj (people* or person* or adult* or m?n or wom?n)).tw. (173989)

21 child/ (1620458)

22 (school* adj2 (pupil* or student*)).tw. (20810)

23 (("11" or "12" or "13" or "14" or "15" or "16" or "17" or "18" or "19") adj (yr? or year?)).tw. (471642)

24 (("11" or "12" or "13" or "14" or "15" or "16" or "17" or "18" or "19") adj4 (old or age?)).tw. (618630)

25 (teen or teens or juvenil*).tw. (87194)

26 or/17-25 (3594742)

27 and/16,26 (28777)

28 ((systematic adj2 review*) or meta-analys* or "meta analysis" or "meta-regression" or "meta regression").ti. (162612)

29 limit 27 to (meta analysis or "systematic review") (290)

30 27 and 28 (271)

31 29 or 30 [srs + self harm+ adolescents] (346)

RCT Search Strategy

Database: Ovid MEDLINE(R) <1946 to July Week 1 2019>

----

1 Self-Injurious Behavior/ (7216)

2 suicide/ or suicide, attempted/ or suicide, assisted/ (57157)

3 Drug Overdose/ (10403)

4 Self Mutilation/ (3181)

5 (selfharm* or selfinjur* or selfinflict* or "self harm*" or "self injur*" or "self inflict*").ti. (4170)

6 ((self or themsel* or onesel*) adj2 (aggress* or harm* or cutt* or immolat* or inflict* or injur* or mutilat* or poison* or damag* or destruct*)).ti. (6433)

7 (auto adj (aggress* or mutilat*)).ti. (73)

8 (automutilat* or "auto mutilation*" or autoaggress* or "auto agress*").ti. (219)

9 suicid*.ti. (36367)

10 (parasuicid* or para-suicid*).ti. (323)

11 ((deliberat* or intentional or intended) adj2 (overdos* or poison* or self poison*)).ti. (368)

12 (poison adj2 (deliberat* or intentional or intended)).ti. (3)

13 (overdos* adj2 (deliberat* or intentional or intended)).ti. (120)

14 NSSI.ti. (35)

15 (headbang* or head-bang*).ti. (65)

16 or/1-15 [self harm] (77902)

17 Adolescent/ (1942456)

18 (teenage* or adolescen* or youth).tw. (261364)

19 young adult/ (753275)

20 (young* adj (people* or person* or adult* or m?n or wom?n)).tw. (151441)

21 child/ (1622676)

22 (school* adj2 (pupil* or student*)).tw. (17529)

23 (("11" or "12" or "13" or "14" or "15" or "16" or "17" or "18" or "19") adj2 (yr? or year?)).tw. (518089)

24 (("11" or "12" or "13" or "14" or "15" or "16" or "17" or "18" or "19") adj4 (old or age?)).tw. (544703)

25 (teen or teens or juvenil*).tw. (77115)

26 or/17-25 [adolescents] (3474833)

27 and/16,26 [self harm and adolescents] (27664)

28 randomized controlled trial.pt. (484695)

29 controlled clinical trial.pt. (93122)

30 randomized.ab. (387592)

31 placebo.ab. (180613)

32 clinical trials as topic.sh. (187532)

33 randomly.ab. (268249)

34 trial.ti. (172865)

35 28 or 29 or 30 or 31 or 32 or 33 or 34 (1107251)

36 exp animals/ not humans.sh. (4595710)

37 35 not 36 [Cochrane RCT precision maximising search filter] (1008831)

38 27 and 37 (1111)

39 limit 38 to english language (1058)

40 38 not 39 (53)

41 limit 38 to yr="2016 -Current" (213)

42 40 or 41 (263)
