# Supplementary material for: Reducing Self-harm in Adolescents. An individual participant data meta-analysis (RISA-IPD): systematic review protocol
Source: BMJ Open. 2021 May 3;11(5):e049255. doi: 10.1136/bmjopen-2021-049255 (PMC8098984; doi:10.1136/bmjopen-2021-049255)
Supplement: Reviewer comments [file bmjopen-2021-049255.reviewer_comments.pdf]

PEER REVIEW HISTORY

BMJ Open publishes all reviews undertaken for accepted manuscripts. Reviewers are asked to complete a checklist review form (**http://bmjopen.bmj.com/site/about/resources/checklist.pdf**) and are provided with free text boxes to elaborate on their assessment. These free text comments are reproduced below.

ARTICLE DETAILS

**TITLE (PROVISIONAL)**	ReducIng Self-harm in Adolescents. An Individual Participant Data meta-analysis (RISA-IPD): Systematic review protocol.
**AUTHORS**	Wright-Hughes, Alexandra; Walwyn, Rebecca; Wright, Judy; Farrin, Amanda; Fonagy, Peter; Ougrin, Dennis; Stahl, Daniel; Cottrell, David

This article was not externally reviewed at BMJ Open. Protocols that have been independently assessed prior to submission to BMJ Open are usually fast-tracked to publication on the grounds that further substantial changes will not be possible. This independent assessment will usually be external, independent review for both a substantial grant award from a non-commercial or government funder and ethics approval.
